# Scrub Typhus Pathogenesis: Innate Immune Response and Lung Injury During *Orientia tsutsugamushi* Infection

**DOI:** 10.3389/fmicb.2019.02065

**Published:** 2019-09-06

**Authors:** Brandon Trent, James Fisher, Lynn Soong

**Affiliations:** ^1^Department of Pathology, University of Texas Medical Branch, Galveston, TX, United States; ^2^Department of Microbiology and Immunology, University of Texas Medical Branch, Galveston, TX, United States

**Keywords:** *Orientia tsutsugamushi*, scrub typhus, acute respiratory distress syndrome, endothelial cell, neutrophil, macrophage

## Abstract

Scrub typhus is an understudied, potentially lethal disease caused by infection with *Orientia tsutsugamushi*. Despite causing an estimated 1 million cases per year and an increasing global presence, mechanisms of scrub typhus pathogenesis remain unclear. One of the most life-threatening conditions that can arise in scrub typhus patients is acute respiratory distress syndrome (ARDS). The development of ARDS is a complex process; some of its pathological hallmarks, including prolonged recruitment of inflammatory immune cells to the lung and vasculature damage, have been observed in humans and/or animal models of *O. tsutsugamushi* infection. Although different cell types and mechanisms may contribute to ARDS development during *O. tsutsugamushi* infection, this review highlights our current evidence of pulmonary endothelial activation and damage, the potential roles of neutrophils and macrophages in the lung, and the knowledge gaps in this field. Continued investigation of the lung microenvironment and cellular interactions will help elucidate disease pathogenesis and possible treatment during scrub typhus.

## Introduction

The etiological agent of scrub typhus is *Orientia tsutsugamushi*, an obligate intracellular bacterium with unique biological features. Unlike other gram negative bacteria, *O. tsutsugamushi* lacks lipopolysaccharides and expresses low levels of unclassical peptidoglycans (Salje, 2017). It has a unique genome, with about 42% of its genetic content consisting of repeat DNA sequences and mobile genetic elements, making genetic manipulation challenging or unsuccessful (Salje, 2017). Despite approximately 1 million new cases every year, scrub typhus is a neglected tropical disease (Paris et al., 2012). The majority of cases occur in the “tsutsugamushi triangle,” a region encompassing much of northern and eastern Asia, islands of the western Pacific Ocean, and a portion of northern Australia (Kelly et al., 2009). However, recent reports have identified cases of *O. tsutsugamushi* infection in areas previously thought free from scrub typhus, such as South America (Weitzel et al., 2016; Xu et al., 2017). Human infection is initiated *via* bite of an infected larval *Leptotrombidium* mite (also known as a chigger). After an incubation period (6–21 days), eschar, fever, headache, and malaise may develop (Kundavaram et al., 2013; Peter et al., 2015). Unless treated quickly, the bacteria can spread systemically, causing interstitial pneumonia, myocardial and hepatic lesions, meningoencephalitis, acute respiratory distress syndrome (ARDS), and multi-organ failure (Peter et al., 2015). Indirect fluorescent antibody (IFA) testing is the diagnostic gold standard and becoming more widely available in high incidence countries; however, IFA must be performed with care due to high-incidence areas having pre-existing immunity to scrub typhus (Blacksell et al., 2007). No vaccine exists for *O. tsutsugamushi,* and poor or limited cross-protection between heterologous *Orientia* strains is of particular concern (Valbuena and Walker, 2012).

Lung infection is common during scrub typhus, and patients typically develop mild interstitial pneumonitis during self-resolving or promptly treated scrub typhus (Song et al., 2004). However, in severe cases, pulmonary pathology includes lung hemorrhage, pulmonary edema, vasculature damage, and diffuse cellular infiltration resulting in ARDS (Jeong et al., 2007; Hsu and Chen, 2008). The development of ARDS is a complex process initiated by direct or indirect lung injury (Longo and Harrison, 2012). Damage to lung endothelial cells (ECs) can lead to accumulation of protein-rich edema in the interstitial and alveolar space, producing characteristic signs and symptoms: progressive dyspnea, hypoxemia, and alveolar infiltrates visible on chest x-ray (Siegel, 2019). Once alternative etiologies are excluded, a patient must meet the following criteria to be diagnosed with ARDS, according to the Berlin definition: respiratory symptoms beginning or worsening within 1 week of an established insult, the presence of bilateral opacities on chest x-ray or CT scan, and moderate-to-severe reduction in oxygenation capacity as measured by the PaO_2_/FiO_2_ fraction (Force et al., 2012). Damage to the vasculature promotes the production of proinflammatory cytokines (i.e., CXCL8 and TNFα) leading to recruitment of leukocytes, especially neutrophils, which secrete their own chemokines and effector proteins, inducing a proinflammatory microenvironment. Without resolution of inflammation and/or prompt treatment, atelectasis and death may occur (Longo and Harrison, 2012).

In scrub typhus, about 6–44% of cases develop ARDS (Wang et al., 2007; Varghese et al., 2013, 2014; Jayasimha et al., 2017). This wide incidence range may be due to different regional strains of *O. tsutsugamushi*, differences in disease detection time, population genetics (Xu et al., 2017), or employment of different diagnostic criteria for ARDS. Both Wang et al. and Jayasimha et al. utilized internationally accepted diagnostic definitions and reported ARDS incidence rates of approximately 11 and 6%, respectively, in scrub typhus patients (Wang et al., 2007; Jayasimha et al., 2017). Publications not stating diagnostic criteria tended to have higher observed incidence rates (Varghese et al., 2013, 2014). Despite these differences, certain risk factors appear to be important for ARDS development during scrub typhus: septic shock, hypoalbuminemia, high circulating white blood cell counts, delayed antibiotic treatment, and advanced age (Tsay and Chang, 2002; Jayasimha et al., 2017). Scrub typhus patients that developed ARDS presented with many clinical features, including pleural effusion, peribronchial thickening, hyaline membrane formation, and occasionally, pulmonary edema (Hsu and Chen, 2008; Abhilash et al., 2016; Sahoo et al., 2016).

Considering the life-threatening complications of ARDS, establishing animal models of this syndrome has garnered significant effort. Murine models of ARDS demonstrate histologic evidence of tissue injury, changes in the alveolar capillary barrier, inflammatory infiltration, and physiologic dysfunction (Aeffner et al., 2015). These models have led to significant advances in understanding the pathophysiology of ARDS; but, there are inherent limiting factors in comparing mouse models of ARDS to humans (Aeffner et al., 2015).

Few studies have investigated scrub typhus-related ARDS, leaving fundamental questions unanswered. We and other groups have recently established a number of murine models of *O. tsutsugamushi* infection mimicking severities of scrub typhus observed in humans (Keller et al., 2014; Shelite et al., 2014; Mendell et al., 2017). Both self-healing and lethal infection models have been established, but pathologies firmly delineating disease outcomes have yet to be uncovered. Murine models, coupled with known human infection data, are now being used to answer questions regarding tissue pathology and immune response during infection. Here, we summarize recent studies regarding pulmonary involvement of several cell types during *O. tsutsugamushi* infection that play key roles in lung injury, focusing on ECs, neutrophils, and macrophages (MΦ). How these three types of immune cells interact with and respond to *O. tsutsugamushi* infection and their contribution to disease progression is critical for the development of effective management strategies.

## Endothelial Cells

ECs are one of the primary cellular targets of *O. tsutsugamushi* during systemic infection. Using autopsy tissues from U.S. soldiers who died of scrub typhus, Moron and colleagues reported *O. tsutsugamushi* in ECs of infected tissues, including the lung (Moron et al., 2001). While *in vitro* evidence exists for *O. tsutsugamushi-*induced activation of human ECs and cell death (Kim et al., 1999; Cho et al., 2001, 2010), there are no detailed reports for the mechanisms determining ARDS development in scrub typhus. It is well documented from other severe diseases that EC activation and damage are hallmarks of ARDS development (Matthay and Zemans, 2011; Gonzales et al., 2015). EC activation can be triggered directly *via* pathogen replication and the recognition of pathogen associated molecular patterns, or indirectly *via* recognition of damage-associated molecular patterns (DAMP) and inflammatory cytokines. EC activation promotes leukocyte adhesion/transmigration, antigen presentation, and cytokine production (Orfanos et al., 2004; Mai et al., 2013). However, uncontrolled EC activation results in excessive influx of neutrophils and MΦs/monocytes to the lung interstitium. This influx leads to increased vascular permeability resulting in edema, tissue hypoxemia, and ARDS (Silliman et al., 2007; Gill et al., 2015; Moussa et al., 2015).

For animal models of scrub typhus, few studies document EC activation and injury. Since 2014, our group has documented pulmonary EC cellular tropism in C57BL/6 mice infected with *O. tsutsugamushi* Karp strain *via* the intravenous (i.v.) (Shelite et al., 2014) or intradermal (i.d.) inoculation route (Soong et al., 2016; Mendell et al., 2017). Regardless of route of inoculation, the lungs carry the higher bacterial loads than the liver, spleen, kidney, and brain (Shelite et al., 2014; Soong et al., 2016; Mendell et al., 2017). Likewise, Keller and colleagues used a footpad inoculation of *O. tsutsugamushi* Karp strain in BALB/c mice and confirmed that the lungs carry the highest bacterial loads. However, bacteria were interpreted to be localized within pulmonary MΦs, rather than in pulmonary ECs (Keller et al., 2014). At present, it is unclear whether *O. tsutsugamushi* preferentially replicates within ECs or phagocytes at different stages in mouse models; future development of reporter bacteria would be valuable in such research.

Sublethal infection in mice *via* i.d. inoculation of *O. tsutsugamushi* Karp, Gilliam, or Woods strains resulted in increased circulation of EC activation markers such as soluble VCAM-1 and soluble ICAM-1 (Sunyakumthorn et al., 2013). The sICAM-1 levels were also significantly increased following i.d. inoculation of rhesus macaques (Sunyakumthorn et al., 2018). Clinical data are consistent with animal models, as sVCAM-1 and sALCAM (soluble activated leukocyte cell adhesion molecule) are increased in scrub typhus patients compared to healthy controls and are correlated with organ dysfunction (Otterdal et al., 2014). However, serum concentration measurements provide little in the context of pulmonary-specific endothelial activation/dysfunction. Use of immunofluorescent staining on infected murine lung tissue would allow visualization of increased EC markers *in vivo* (Kim et al., 2016). More importantly, *O. tsutsugamushi*-infected, lung-derived cells would be of great value for flow cytometric analyses of alterations in EC-specific markers and EC viability or gene profile analyses, as in other infection models (Singer et al., 2016; Lai et al., 2018; Lukowski et al., 2019).

Given the evident EC activation, it is speculated that *O. tsutsugamushi* infection can trigger the expression/release of DAMP molecules, and our studies with infected human ECs and mouse tissues suggest that a pathogenic role of IL-33 (Shelite et al., 2016), a nucleus-located alarmin, belongs to the IL-1 family. It is known from other model systems that IL-33 released from damaged ECs can be processed and activated extracellularly *via* cleavage by enzymes such as neutrophil elastase or cathepsin G (Lefrancais et al., 2012), acting as a DAMP molecules on nearby EC (Cayrol and Girard, 2009; Gautier et al., 2016). IL-33^−/−^ mice demonstrated significantly less weight loss during lethal infection with *O. tsutsugamushi*, and exogenous recombinant IL-33 treatment had exacerbated disease following sublethal infection (Shelite et al., 2016). Complementing these findings, neutralizing IL-33 antibody was shown to reduce inflammation and lung injury in the murine LPS-induced ARDS model (Lin et al., 2016). Currently, there is no evidence of IL-33 production in pulmonary ECs or alveolar epithelium in *O. tsutsugamushi*-infected mice. The utilization of IL-33 reporter mice (Heyen et al., 2016) will help examine cellular sources and kinetics of IL-33 production correlating to local pathology during *O. tsutsugamushi* infection and disease pathology.

For EC-related biomarkers in severe *O. tsutsugamushi* infection, angiopoietin 2 (Ang2) and angiopoietin 1 (Ang1) levels and their expression ratio are highly relevant (van Meurs et al., 2009; Parikh, 2017). While Ang1 is constitutively expressed by pericytes to promote EC quiescence and effective barrier function (van Meurs et al., 2009), Ang2 is released/produced by ECs during activation or damage. Both molecules compete to bind Tie2 in order to modulate EC function (van Meurs et al., 2009). There are reports that increased Ang2 levels correlate with ARDS development in malaria (Ghosh et al., 2016; Higgins et al., 2016), influenza (Ghosh et al., 2016), and sepsis (David et al., 2011; Stiehl et al., 2014; Ghosh et al., 2016). Our *O. tsutsugamushi-*infected mouse models have consistently shown an increased Ang2/Ang1 mRNA ratio in the lung, liver, and brain tissues at the onset of disease and prior to host death (Soong et al., 2014, 2017). Whether tissue and serum levels of Ang2/Ang1 correlate with human scrub typhus patients has yet to be determined.

Our current understanding of *O. tsutsugamushi* infection-induced EC activation or injury is limited, however, by examining gene expression of primary human EC cultures and mouse lung tissue homogenates (Soong et al., 2014; Shelite et al., 2016). Studies to determine changes in pulmonary endothelium during infection would benefit from more honed approaches, such as the use of single-cell RNA sequencing to characterize pathways important to both EC activation and homeostasis.

## Neutrophils

Neutrophils are the most abundant leukocyte in circulation and are considered the “first responders” during tissue injury or infection (Summers et al., 2010). Neutrophil recruitment plays an important function in early control of human pathogens (Bou Ghanem et al., 2015; Witter et al., 2016), but prolonged neutrophil involvement can be detrimental to host health (Saffarzadeh et al., 2012; Bou Ghanem et al., 2015). Neutrophil effector functions include phagocytosis and killing of bacteria, release of antimicrobial granule contents, and formation of neutrophil extracellular traps (NETs). The latter two functions work to combat pathogens but can also damage host tissues (Grommes and Soehnlein, 2011). In other model systems, NET formation in the lung is known to promote ARDS development, partially *via* increased epithelial and endothelial cytotoxicity (Saffarzadeh et al., 2012). Mice treated with DNase I or deletion of the *PADI4* gene have reduced lung injury in a murine model of bacterial pneumonia-induced ARDS, at least in part due to reduced NET formation (Lefrançais et al., 2018).

Few studies have addressed the role of neutrophils during scrub typhus. Early *in vitro* work documented the presence of *O. tsutsugamushi* in phagosomes within neutrophils, as well as in the extracellular space (Rikihisa and Ito, 1982). However, despite such findings nearly 40 years ago, it remains unclear if neutrophils are capable of killing the engulfed bacterium or if neutrophils provide another cellular niche for bacterial growth. It is known that neutrophils are detected at the site of the chigger bite in humans and that neutrophilia occurs during acute infection (Cho et al., 2012; Paris et al., 2012). Scrub typhus patients have increased CXCL8, a potent neutrophil chemoattractant, and its serum levels correlated with disease severity and mortality (Astrup et al., 2014). CXCL8 is increased in individuals with ARDS and is believed to contribute to ARDS pathology *via* neutrophil recruitment, inhibiting neutrophil apoptosis and activating lung endothelium (Chollet-Martin et al., 1993; Allen and Kurdowska, 2014). Importantly, Paris et al. showed that markers of neutrophil activation and possible NET formation were also significantly upregulated in the plasma of severe scrub typhus patients compared to less severe scrub typhus cases (Paris et al., 2015; Park et al., 2018).

Neutrophil activation and pulmonary recruitment during *O. tsutsugamushi* infection have been primarily studied in murine models. Immunohistochemical studies in both footpad and i.v. infection models revealed neutrophil infiltration of the pulmonary interstitium (Keller et al., 2014; Soong et al., 2014). Lung tissues from infected mice are strongly positive for myeloperoxidase (MPO; Soong et al., 2014), a peroxidase enzyme abundantly present in neutrophil primary granules that act as an antimicrobial. Since mice i.v. inoculated with *O. tsutsugamushi* have marked increase in *CXCL1* and *CXCL2* transcripts at the onset of disease (day 6) and prior to host death (Soong et al., 2014), the use of gene-targeted knockout mice, as well as specific inhibitors or antibodies to block these chemokines or CXCR2, will help dissect protective versus pathogenic role of neutrophils in scrub typhus (Sônego et al., 2016). More research is needed to examine *O. tsutsugamushi* killing or growth within neutrophils; such information will elucidate bacterium-neutrophil interactions and their contribution to bacterial control versus dissemination. The contribution of specific neutrophil processes (e.g., degranulation and NET formation) to lung injury in scrub typhus should be investigated.

## Macrophages

Both resident alveolar MΦs and recruited circulating monocytes are known to play important roles in the development and resolution of ARDS in other infections (Huang et al., 2018). For example, *in vivo* depletion models have demonstrated that MΦs can play either a pathogenic or a protective role during ARDS, depending on the method of lung injury induction and time of disease progression (Broug-Holub et al., 1997; Eyal et al., 2007; Narasaraju et al., 2011). MΦs recruited to the lungs during ARDS have been shown to adopt an initial M1 profile, generating an acute inflammatory response and combating bacterial pathogens (Benoit et al., 2008; Aggarwal et al., 2014; D’Alessio et al., 2016). M1 polarization is believed to be partially responsible for initiation of ARDS *via* secretion of inflammatory cytokines and chemokines and production of reactive oxygen and nitrogen species (Aggarwal et al., 2014; Lu et al., 2018). Shifting the pulmonary MΦ population from M1 to an alternative M2 phenotype is understood to be important for resolution of lung inflammation and tissue healing (Aggarwal et al., 2014; Vergadi et al., 2014; D’Alessio et al., 2016).

During *O. tsutsugamushi* infection, MΦs can serve not only as a cellular target of bacterial replication but also as an inflammatory response initiator (Cho et al., 2000; Moron et al., 2001). *In vitro* infection of MΦs by *O. tsutsugamushi* can trigger inflammasome activation (Koo et al., 2012), initiate inflammatory signaling cascades (Yun et al., 2009), and generate chemokines for recruiting T cells, neutrophils, and monocytes (Cho et al., 2000). In humans (Paris et al., 2012) and rhesus macaque (Sunyakumthorn et al., 2013), infection of MΦs is observed early in eschars and may propagate lymphogenous dissemination.

The recruitment and role of pulmonary MΦs during systemic *O. tsutsugamushi* infection have been investigated using various murine models of infection. Self-resolving footpad inoculation generates a systemic infection with recruited MΦs in the pleura and bronchial alveolar lymphatic tissue (Keller et al., 2014). Furthermore, *O. tsutsugamushi* was present in interstitial MΦs, and iNOS (inducible nitric oxide) production was induced in the lung following infection (Keller et al., 2014). In congruence with animal findings, iNOS production has been observed in interstitial MΦs from patients who succumbed to ARDS during severe scrub typhus (Hsu and Chen, 2008). Generation of iNOS to combat invading pathogens is a product of MΦ activation and is associated with M1 polarization. iNOS inhibition in mouse macrophages was associated with increased macrophage bacterial loads (Keller et al., 2014). The role of iNOS production in MΦ control of *O. tsutsugamushi* is still somewhat uncertain, however, as others have reported increased bacterial loads within MΦ during prolonged infection that appears linked to iNOS production (Ogawa et al., 2017). Evidence of inflammatory M1 polarization is confirmed by transcriptome analysis of human monocytes and peripheral blood mononuclear cells from scrub typhus patients (Tantibhedhyangkul et al., 2011). While M1 polarization is known to contribute to lung injury and ARDS development (Aggarwal et al., 2014), the contribution of M1-polarized MΦs to lung injury during scrub typhus is still unclear. Furthermore, the presence/role of M2 MΦs during *O. tsutsugamushi* infection has not been explored. Future studies classifying MΦ subsets present in the lung and identifying their contribution to inflammation and tissue health during scrub typhus will be important to understand disease pathogenesis. Use of *in vivo* models with conditional knockout genes in MΦs for iNOS production (McNeill et al., 2015) or specific M2-type genes, such as arginase 1 (Suwanpradid et al., 2017), may help us understand the role of MΦ polarization in bacterial dissemination and disease progression.

## Concluding Remarks

**Figure 1:**
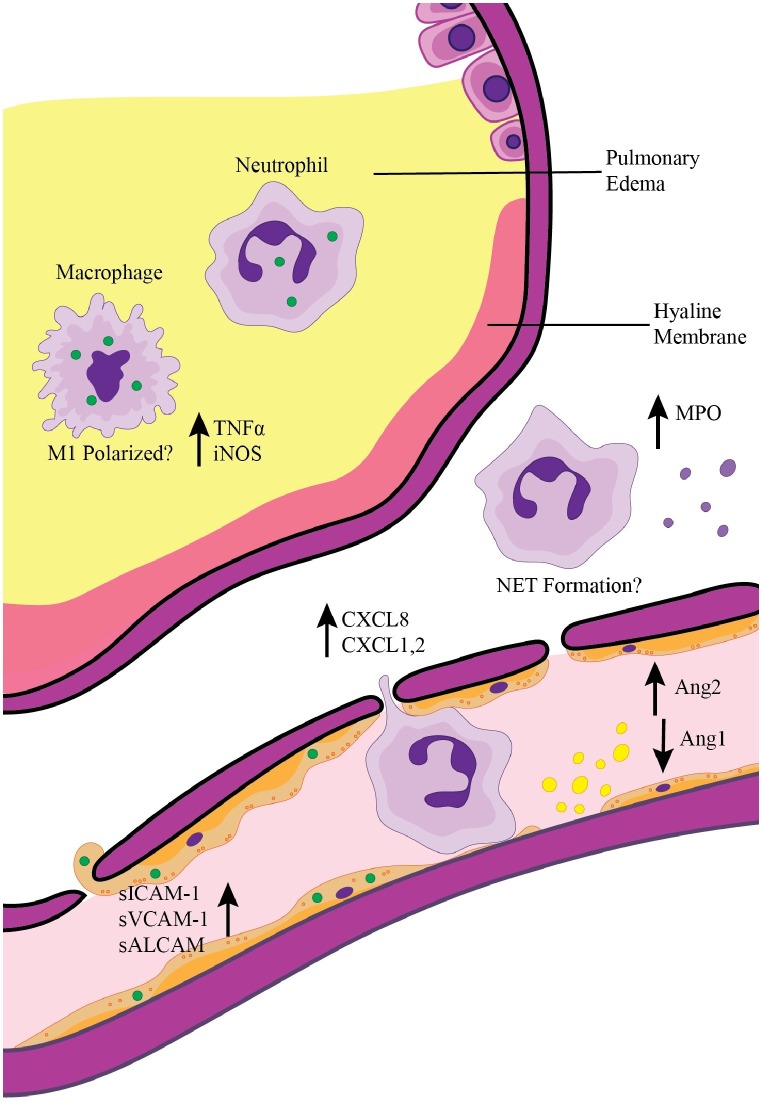
Pulmonary inflammation and ARDS development during scrub typhus. Activation of pulmonary endothelial cells *via* direct infection with *O. tsutsugamushi* (green circles) or by interaction with inflammatory cytokines leads to the release of Ang2 and upregulation of adhesion molecules. Endothelial activation results in recruitment of neutrophils and macrophages, which can in turn secrete chemokines for further immune cell recruitment (i.e., CXCL1, CXCL2, and CXCL8). Infection of endothelial cells coupled with continued recruitment of inflammatory immune cells results in breakdown of the endothelial barrier, pleural effusion, and pulmonary edema seen in severe scrub typhus patients. Activation of recruited neutrophils and macrophages contributes to the inflammatory pulmonary environment.

Although considered a neglected pathogen, *O. tsutsugamushi* infection and disease burden in endemic areas have become better appreciated in recent years (Bonell et al., 2017). Life-threatening complications like ARDS can quickly arise in scrub typhus patients not treated promptly. Therefore, it is important to understand mechanisms contributing to ARDS development during *O. tsutsugamushi* infection. This review highlights what is known regarding vascular activation and the innate immune response during scrub typhus (Figure 1). While adaptive immune cell subsets (CD4^+^ and CD8^+^ T cells and Foxp3^+^ regulatory T cells) are not illustrated in Figure 1, their roles in *O. tsutsugamushi* infection need to be examined, given their important roles in ARDS development in other infection models (Adamzik et al., 2013; Risso et al., 2015; Li et al., 2016). Murine models of scrub typhus will help dissect key immune components in controlling *O. tsutsugamushi* infection and host factors and mechanisms underlying cellular injury and ARDS development in severe scrub typhus.

## Author Contributions

BT conceived and drafted the manuscript and conceived and organized the figure. JF and BT revised and edited the manuscript. LS revised and edited the manuscript and figure.

### Conflict of Interest Statement

The authors declare that the research was conducted in the absence of any commercial or financial relationships that could be construed as a potential conflict of interest.
